# Extended-release Injectable Buprenorphine Initiation in the Emergency Department

**DOI:** 10.5811/westjem.21299

**Published:** 2025-07-12

**Authors:** Brittany Cesar, Jessica Moore, Raluca Isenberg, Jessica Heil, Rachel Rafeq, Rachel Haroz, Matthew Salzman, Alice V. Ely

**Affiliations:** * Cooper University Health Care - Center for Healing, Department of Addiction Medicine, Camden, New Jersey; † Cooper University Health Care - Cooper Research Institute, Camden, New Jersey; ‡ Cooper University Health Care, Department of Emergency Medicine, Camden, New Jersey; § Cooper Medical School of Rowan University, Camden, New Jersey

## Abstract

**Introduction:**

Extended-release buprenorphine (XR-BUP) is a long-acting injectable medication used for the treatment of opioid use disorder (OUD). It is currently approved for use in patients who have been administered at least seven days of sublingual buprenorphine (SL-BUP). For patients with OUD who are unstable (ie, not at treatment goal, with active opioid use) or not yet on medication for OUD (MOUD) such as SL-BUP, the emergency department (ED) setting is an essential location for access to treatment. There is, as yet, no research on the utility of on-demand XR-BUP administration in the ED.

**Methods:**

We performed a retrospective cohort study of individuals with OUD who received XR-BUP in the ED through our novel reallocation pathway. We reviewed charts from an addiction medicine specialty outpatient clinic to determine retention in treatment, continuation on XR-BUP, and reported quantitative analysis. Our primary outcome was retention in treatment, measured by subsequent XR-BUP injection after initial ED XR-BUP administration. The secondary outcome was the reason for ED administration of XR-BUP (as opposed to administration in the clinic setting).

**Results:**

Our study population included 69 patients (68.2% male). Our primary outcome showed that 51 (73.9%) patients who had their first injection in the ED received a second XR-BUP injection and 40 (58%) received their third XR-BUP injection. Our secondary outcome showed that 7.2% had barriers with access to treatment; however, most of the patients received the injection due to instability of the treatment of the OUD (69.6%). These patients were either unable to adhere to MOUD, reported issues with the prescription, or were still using substances while on MOUD. For 52 (75%) patients, the index ED injection was their first ever XR-BUP injection. Logistical regression analyses demonstrated that clinical and demographic factors did not lead to increased attrition, while patients with other co-occurring substance use disorders were more likely to present for follow-up treatment.

**Conclusion:**

In our retrospective study, patients who received ED-initiated extended-release buprenorphine had a strong retention rate compared to previous studies evaluating ED-initiated sublingual BUP (retention rates ranging from 16.7–60%). The ED provided a convenient healthcare access point for XR-BUP initiation. The XR-BUP is a helpful tool for achieving induction after failed SL-BUP initiation and may have further implications in minimizing treatment gaps after discharge and improving OUD treatment retention.

## INTRODUCTION

Opioid use disorder (OUD) is highly prevalent in the United States, with considerable associated morbidity and mortality.^1^ Buprenorphine, a high-affinity partial μ-opioid agonist, is an effective medication for OUD (MOUD) that treats opioid cravings and withdrawal. There is abundant data demonstrating buprenorphine’s efficacy in reducing mortality for patients with OUD.^2^–^4^ Extended-release buprenorphine (XR-BUP) is an injectable formulation with a duration of action of four weeks. It is currently approved by the US Food and Drug Administration as an alternative treatment option for OUD in patients who have been stable on sublingual buprenorphine (SL-BUP) for at least seven days.^5^–^7^

The XR-BUP is an appealing formulation for patients who desire the convenience of once-monthly administration over sublingual medications, which require 15–20 minutes of administration of a dissolvable tablet or film taken 1–4 times per day.^8^ Along with the convenience of XR-BUP, like most extended-release injectable medications, it is postulated to contribute to greater medication efficacy due to increased adherence. The XR-BUP provides a more continuous, steady-state level of buprenorphine^8^; thus, it confers relative protection from overdose for a critical one-month period. The XR-BUP has been associated with improved treatment retention rates in patients in other settings.^9^–^12^

Despite the benefits of XR-BUP, patients who desire this medication can face multiple barriers to receiving it, including insurance coverage, cost, and access to a clinician who administers it. Compared to SL-BUP, XR-BUP is less widely available to patients, substantially more expensive, and less likely to be covered by insurance.^13^ Initiating XR-BUP is often a 1–6 week process during which the medication must be ordered from a specialty pharmacy, delivered to a clinic, and then administered at a subsequent encounter.^14^ This process generally precludes same-day or on-demand administration of XR-BUP; however, through a collaboration with our addiction medicine division, the emergency department (ED), our institution’s inpatient pharmacy, and a New Jersey Medicaid managed care organization, we created a novel alternate pathway to make XR-BUP available for patients in our ED.^15^ This reallocation process transferred unused XR-BUP from our outpatient addiction medicine clinic to the institution’s inpatient pharmacy. Patients in the ED were candidates if they had a specific NJ Medicaid managed care organization.^15^ Emergency clinicians were informed of this process and used the addiction medicine consult service to counsel patients and administer the medication.

Given the long duration of action of XR-BUP, this MOUD formulation is of particular value for treatment retention.^16^ Several demographic, psychosocial, and comorbidity factors impact retention in OUD treatment, including age,^17^ sex,^18^ race,^19^ housing stability,^17^,^19^,^20^ insurance,^21^ and use of other substances,^17^ as well as comorbid psychiatric or medical issues.^20^,^22^,^23^ Notably, these studies typically assessed retention in MOUD treatment with SL-BUP, but none specifically examined patients administered XR-BUP in the ED. It is critical to determine whether treatment retention on XR-BUP is related to sociodemographic and medical variables to identify and coordinate care for potentially vulnerable patients.

**Population Health Research Capsule**

What do we already know about this issue?

*Extended-release buprenorphine (XR-BUP) is a long-acting injectable medication used for the treatment of opioid use disorder (OUD)*.

What was the research question?

*Does use of ED-initiated XR-BUP increase retention in treatment of OUD?*

What was the major finding of the study?

*Retention in treatment at 60 and at 90 days was 73.9% and 57.9%, respectively*.

How does this improve population health?

*Administering XR-BUP in the emergency department is a possible strategy for improving outcomes for treatment of OUD*.

Our aim in this retrospective cohort study was to characterize patients who received XR-BUP in the ED and identify predictors of treatment retention. Our primary outcome for measuring retention was subsequent XR-BUP injections after initial ED XR-BUP administration. Reason for ED-administration of XR-BUP was also of interest as an exploratory descriptive variable. To our knowledge, there have been no previously published studies evaluating outcomes for patients administered on-demand XR-BUP in an ED setting. We hypothesized that most patients received XR-BUP in the ED due to lack of access and/or instability of OUD. We hypothesized that once initiating XR-BUP, most patients would receive follow-up injections of XR-BUP. We further hypothesized that medical, psychiatric, and co-substance use-related comorbidities at baseline would influence likelihood of treatment retention.

## METHODS

### Setting

Cooper University Hospital (CUH) is a Level I trauma center and academic, tertiary referral hospital located in Camden, NJ, with 635 inpatient beds. The ED, which has over 64 beds and an annual volume of over 80,000 visits, serves as a training site for an emergency medicine residency program. The hospital has an inpatient addiction medicine consult team and low-barrier addiction medicine outpatient specialty clinics. This study was approved by the CUH Institutional Review Board (IRB protocol #22-271).

### Study Population

A total of 69 patients (68.2% male; mean age 40.16 years (SD +/− 9.66)) who received XR-BUP in the ED were included in the study. Patients were identified by an ED pharmacist as anyone who received an EX-BUP injection in the ED. Patients were included if they were ≥18 years of age, had a documented diagnosis of OUD, and received their index XR-BUP injection in the CUH ED between December 2018–December 2022. We included patients who were on buprenorphine maintenance therapy and those not on MOUD prior to index injection. There was no standard protocol for patient selection (given this was a retrospective review); however, the most common process was as follows: an emergency clinician identified a consentable patient with OUD with active use who was interested in XR-BUP (all 300 milligram (mg) doses). The emergency clinician consulted an addiction medicine specialist who, in collaboration with the ED team, counseled the patient on the risks (most often risk of precipitated withdrawal) and benefits of the medication. If the patient agreed, they received the medication. Monitoring in the ED was not required after injection. There were few instances where emergency clinicians administered the XR-BUP without consult with addiction medicine. Exclusion criteria included incarceration, pregnancy, and intoxication. After injection, patients were directed to make a follow-up appointment with the outpatient addiction clinic of their choice. If the patient desired to follow up with our addiction clinic, a message was sent to a transitional navigator to reach out to the patient to schedule the appointment.

### Data Collection

The elements of optimal retrospective chart review are included in this section.^24^

Using addiction medicine specialist feedback, we created a data collection tool and abstraction manual using Research Electronic Data Capture (REDCap) tools hosted at CUH. Incomplete data was mitigated by performing a pilot test on the abstraction form. Authors BC and AE pilot-tested the collection tool and manual by first abstracting 10 records, which were then adjusted according to the findings of the pilot test. Three research assistants (RA) assisted with data abstraction. The RAs were not blinded to the hypothesis. They were trained using the abstraction manual and then assigned five records to abstract from the electronic health record (EHR). (Both the ED and the clinic used the institutional EHR system [Epic Systems Corporation, Verona, WI]). If, after record review, zero discrepancies were found, the RA continued abstracting. If any discrepancies were found, the RA was retrained and assigned five more records to abstract. This process continued until the RA had zero discrepancies. They were randomly assigned a section of the dataset (with overlap for double abstraction review).

We did not formally test inter-rater reliability; however, a research coordinator (who was not involved in data abstraction) reviewed the first 10 double-abstracted data from each RA to ensure the abstracted data were congruent. The data were congruent.

### Analysis

We derived all data via retrospective chart inquiry and analyzed it using RedCap; the data was de-identified prior to statistical analysis. Analyses were conducted in SPSS v29 (SPSS Statistics, IBM Corp, Armonk, NY). We conducted descriptive analyses to characterize the patient population in terms of demographic variables, social determinants of health, medical and psychiatric comorbidities, and treatment outcome variables. No patients were excluded in the descriptive analyses. We used logistic regression to determine the relationship of variables to treatment retention vs attrition as measured by receipt of follow-up XR-BUP injections. We also calculated confidence intervals (CI) at 95%. Analysis of variance (ANOVA) was used to compare demographic groups on medical, psychiatric, and treatment outcome data.

We used logistic regression models to analyze the relationship of descriptive variables (sex, race, ethnicity, insurance type, housing stability), clinical variables (number of prior XR-BUP injections, number of ED visits in the prior six months), and medical variables (presence of medical, psychiatric, or substance use disorder comorbidities) while controlling for age, with whether the patient was still in treatment at one- and three-month follow-ups.

Three patients were excluded as outliers for the number of ED visits in the prior six months, two for number of prior XR-BUP injections (only logistic regression model). Race, ethnicity, and insurance type had incomplete information, with too few patients in some categories. Race was converted to a dichotomous variable (White vs non-White) to create more parity in group size, while we excluded ethnicity and insurance type from the final model given that group size was still too uneven for valid analyses. Sex, dichotomized race, housing, and comorbidity variables were each examined in univariate models, while clinical variables were included in one combined model.

## RESULTS

### Descriptive Analyses

#### Demographics

The 69 patients (68.2% male; mean age 40.16 years [SD +/− 9.66]) who received XR-BUP in the ED were included in the study demographic and medical outcome data for the current analyses. Scores for variables >3 SD from the mean for each group were excluded from analyses and are noted below. Demographic variables are described in Table 1; however, it should be noted that 87% of the patients had Medicaid insurance, and almost 5% had no insurance. More than a third of the patients (36.2%) had unstable housing.

The vast majority (84%) of the patients had a comorbid psychiatric diagnosis, anxiety being the most frequent diagnosis reported (59%), followed by depression and bipolar disorder (57.9% and 42%, respectively). A total of 88% of patients had a comorbid substance use at the time of the index injection, of which cocaine was the most preferred (76%). See full list of comorbidities in Table 2.

#### Substance Use Patterns

The most commonly reported substance use method at the time of index injection was intranasal by 31 patients 44.9%), followed by intravenous (29, 42.0%), oral (1, 1.4%) and smoking (1, 1.4%). The majority of patients reported primary substance use of heroin/fentanyl (66, 95.7%), rather than prescription opioids (3, 4.3%). Sixty-one patients (88.4%) were noted to have comorbid substance use at the time of index injection, while eight (11.6%) did not. These other substances included the following: alcohol (9, 14.5%); amphetamines (1, 1.6%); benzodiazepines (24, 38.7%); crack cocaine/cocaine (53, 85.5%); marijuana (cannabis, THC) (19, 30.6%); methamphetamine (11, 17.7%); and PCP (4, 6.5%). Of note, tobacco was not included in comorbid substance use.

#### Treatment Outcomes

For our primary outcome, retention in treatment at 60 and 90 days was 73.9% and 57.9%, respectively. This includes patients who received follow-up injections in the outpatient clinic in addition to those who returned to the ED for their subsequent doses. Of those patients following up at 30 days, 53% received their second XR-BUP injection in the outpatient setting; at 60 days, 47.8% of the patients received their third XR-BUP injection in the outpatient setting. The most frequent reasons for ED-initiated XR-BUP administration were as follows: instability of treatment of OUD, such as frequently missed visits, medication non-adherence, or continued illicit opioid use (69.6%); patient preference (15%); access challenges (incarceration, entering residential treatment that did not allow MOUD, could not get appointment) (7.2%); prescription issues (4.3%); unknown (1.4%); and other (1.4%).

For most patients (75%), the ED index injection was their first ever XR-BUP injection. Those who had received prior injections had a mean of four XR-BUP administrations prior to the ED index injection (range 1–20). Most patients (97.1%) had not undergone buprenorphine micro-induction (a process of starting low doses of buprenorphine and increasing daily to therapeutic maintenance dosing of buprenorphine while simultaneously given opioid agonists) prior to the index XR-BUP injection.

Mean time from first addiction medicine clinic contact to index injection (counted as patient’s first addiction medicine visit) was 67.77 weeks (SD 0–208 weeks). From chart inquiry, 11.6% of patients reported adverse reactions to XR-BUP injection. Of this 11.6%, adverse injection reactions included pain, swelling, itching, rash, redness, or infection (37.5% of 11.6%); withdrawal symptoms (37.5%); and other (25%). Sixty-one patients (88.4%) received a supplemental script for SL-BUP, and eight (11.6%) did not. The daily dose of SL-BUP patients continued taking following their index injection ranged from 8–24 mg total daily dose (mean 19.28 mg, SD 5.73).

### Logistic Regression Analysis

#### Predictors of treatment retention

##### Demographic variables

Univariate logistic regression models predicting receipt of the second dose of XR-BUP (with age as a covariate) based on housing status or dichotomized race were not statistically significant. The model that regressed second dose on sex, controlling for age, was significant (χ^2^ (2)= 7.5, *P* = .02), but main effect of patient’s sex had only a trend-level relationship with odds of retention at one month (B = 1.1, *P* =.07, Exp(B) = 2.9, 95% CI 0.9–9.3). None of the demographic variable models significantly predicted odds of retention at three months.

##### Clinical variables

The logistic regression model for receipt of the second dose of XR-BUP regressed on the number of prior XR-BUP injections and the number of ED visits in the prior six months while controlling for age was not statistically significant. The model predicting odds of retention at three months was also not significant.

##### Comorbid diagnoses

Univariate logistic regression models predicting receipt of the second dose of XR-BUP (controlling for age) based on medical comorbidity was not significant. The psychiatric comorbidity model was statistically significant (χ^2^ (2)= 7.1, *P* = .03) but the main effect of psychiatric comorbidity had only a trend-level relationship with odds of one-month retention (B = 1.3, *P* = .08, Exp(B) = 3.6, 95% CI 0.8–15.0). In contrast, the model for additional substance use disorder comorbidity was significant (χ^2^ (2)= 12.2, *P* < .001), and it was found that the odds of retention after one month were significantly increased for patients who had a diagnosis of an additional comorbid substance use disorder or dependence (B = 2.0, *P* < .001, Exp(B) = 7.5, 95% CI 1.8–31.8). At three-month follow-up, odds of retention in treatment remained higher for patients with additional substance use comorbidities, with the model and main effect significant (χ^2^ (2) = 6.1, *P* = .05; B = 1.6, *P* = .02, Exp(B) = 4.8, 95% CI 1.2–19.1). Models predicting retention based on medical comorbidity and psychiatric comorbidity were both non-significant.

## DISCUSSION

To our knowledge, this study is the first to evaluate on-demand XR-BUP administration in the ED setting and identify predictors of retention. The ED can serve as a critical access point for MOUD initiation, especially in unstable patients who are actively using illicit substances and having trouble with BUP initiation. Although XR-BUP is currently approved only for use with patients stable on SL-BUP for at least seven days, almost all the patients in this study received their index XR-BUP injection in the context of instability of OUD (ie, difficulty with outpatient SL-BUP initiation). This suggests that on-demand, ED-initiated XR-BUP may be a safe and effective alternative treatment strategy for patients with unstable OUD. The ED is commonly used for stabilization of acute illness in other diseases where patients are not linked with continuity of care (ie, psychiatric illness); our findings suggest this utility can be extended to SUD.

Notably, the retention rate demonstrated in this study was considerably higher than that found in similar studies looking at 30-day follow-up after ED-initiated SL-BUP (43.1–54.1%).^25^,^26^ This outcome is especially striking when taking into account that our studied patient population had appreciable housing insecurity, co-substance use, and medical comorbidities. We suspect this increased treatment retention may be attributed to the long-acting, extended-release nature of the medication, which maintains steady-state blood levels of buprenorphine for several weeks, thus, reducing burden on patients.

Ad hoc analyses of predictors of retention at one and three months suggest that demographic variables, social determinants of health such as housing stability, and comorbid medical conditions may not increase risk of dropout from treatment in this population, which further encourages flexibility in prescribing. Of particular note, those who had a co-occurring substance use disorder were more than seven times as likely to present for their second shot compared to those who had no comorbid substance use disorder, and more than four times to still be in treatment at three months. It is possible that this population of patients with multiple substance use disorders may be particularly responsive to XR-BUP, may have less severe OUD, or may benefit more markedly from the added stability of injectable MOUD. Future research exploring neurobiological or metabolic correlates of XR-BUP in those with multiple SUD diagnoses would be useful to determine the potentially unique benefit to these patients.

Future research is necessary to fully describe the role of ED-initiated XR-BUP in improving long-term outcomes. Larger and prospective studies are needed to evaluate the effectiveness of ED-initiated XR-BUP in improving treatment retention, adherence, and abstinence. Next steps should also examine the effect of ED-initiated XR-BUP on ED utilization, rates of opioid overdose, and mortality rates. Further investigations may focus on the cost-effectiveness of ED-initiated XR-BUP. While it is an expensive medication, we suspect that over time its use could offset significant healthcare costs related to continued opioid use. The intent of this reallocation program was to reduce costs by repurposing an expensive medication that would otherwise go to waste; a cost-benefit analysis could favor the addition of XR-BUP to hospital formularies in the future. Finally, qualitative studies may also be helpful in assessing the patient perspective and relationship of XR-BUP in facilitating more prolonged recovery.

## LIMITATIONS

While use of XR-BUP in this study was technically off-label, more rapid initiation of XR-BUP has become increasingly common in practice, and small studies have characterized patients receiving the medication in this manner.^27^,^28^ Generally, the primary concern with premature initiation of XR-BUP is precipitated opioid withdrawal syndrome; in the event that a partial μ-opioid agonist (BUP) is administered while a patient has high blood levels of full μ-opioid agonist (such as fentanyl), the partial μ-opioid agonist will displace the full μ-opioid agonist, quickly inducing a severe opioid withdrawal syndrome.^29^ Of this studied population, many were “unstable” and possibly willing to accept the risk of short-term precipitated withdrawal for the anticipated benefit of long-term future stability on a maintenance medication. Although precipitated withdrawal is a feared side effect for patients, it is not inherently lethal, and must be weighed with the generally greater risk of morbidity and mortality in this population that is associated with continued use of illicit opioids.^2^ Patients were prescribed SL-BUP supplemental scripts to compensate for anticipated opioid tolerance (as it often takes multiple injections to reach steady state) as well as mitigate any possible post-injection precipitated withdrawal symptoms.

Another limitation is that this study did not have a standard protocol for the emergency clinician’s patient selection; however, aside from a few instances overnight, most patients were counseled by a specialist from the addiction consult team. Many emergency clinicians are not acquainted with the process by which patients could be candidates for XR-BUP. We did not use an objective marker for stability of OUD, such as buprenorphine/norbuprenorphine urine toxicology levels, nor did we disclose a subjective marker of current opioid withdrawal. With this in mind, it would be advantageous to have additional training for emergency physicians with regard to candidate selection and patient counseling if a program akin to that described in our study were to be implemented elsewhere. The ED-addiction consultant collaboration we describe in this study may further limit generalizability to the wider OUD population.

The analysis was limited to a small sample size of patients in a single ED, with one payor and that followed up in a single specialty addiction clinic. Thus, our patient population characteristics may limit the generalizability of our findings to other settings. The cohort of patients receiving XR-BUP were candidates due to the reallocation program with a managed care organization; XR-BUP was not available to other patients in our ED due to insurance restrictions.

An additional limitation to the generalizability of this study is the pre-existing reallocation program for administration of XR-BUP in the ED, which is relatively unique. There are significant logistical barriers involved with ordering XR-BUP in the outpatient setting; thus, it is too expensive to be kept on formulary in most inpatient pharmacies. In the setting for this study, we already had an extensive outpatient XR-BUP program that provided excess unused product that could be allocated for ED or inpatient use. Given the success of retaining patients in treatment using this approach, our findings support the utility of this reallocation program.^8^

Finally, this study was retrospective in nature, and we did not compare treatment retention rates for XR-BUP to patients in this population with other ED-initiated MOUD (such as SL-BUP).

## CONCLUSION

This retrospective cohort study highlights the feasibility of administering extended-release buprenorphine in an ED setting and demonstrates its potential implications for access and cost barriers. Our findings showed that ED-initiated XR-BUP was associated with improved retention rates, increased from rates pertaining to ED-initiated sublingual buprenorphine.^9^ Furthermore, clinical and demographic variables did not lead to increased attrition, supporting the use of XR-BUP in patients with complex presentations. The ED provided an important and convenient healthcare access point for XR-BUP initiation. The XR-BUP may be a helpful tool for achieving induction after failed SL-BUP initiation and can minimize treatment gaps after discharge and improve retention.

Future research will focus on evaluation of the impact of XR-BUP on ED utilization, patient-centered outcomes, and morbidity and mortality related to opioid use disorder. Clinical goals will support training our emergency clinicians for counseling and administration of this medication. Despite the many barriers to this medication, XR-BUP is an effective alternative treatment strategy for patients with unstable opioid use disorder. The ED is an essential setting for initiation of medication for OUD and a critical access point for unstable patients.

## Figures and Tables

**Table 1:** Study participant demographics in a study of extended release buprenorphine initiation in the emergency department.

Mean age	40.16	
Sex		
Male		68.2%
Female		31.8%
Race		
Asian	1	1.4%
Black	20	29%
White	41	41.6%
Hispanic	5	7.2%
Unknown	2	2.9%
Ethnicity		
Hispanic	10	14.5%
Non-Hispanic	59	85.5%
Insurance		
Medicaid	60	87%
Medicare	6	8.7%
Private	0	0
None	3	4.3%
Housing		
Stable	41	59.4%
Unstable	25	36.2%
Not documented	3	4.3%

**Table 2:** Study participant comorbidities in a study of extended release buprenorphine initiation in the emergency department.

Comorbidities		
Medical		
Congestive heart failure	4	5.7%
CVA without hemiplegia	1	1.4%
Ulcer disease	6	8.7%
Mild liver disease	2	2.9%
Diabetes without complications	3	4.3%
Connective tissue disease	3	4.3%
Chronic obstructive pulmonary disease	21	30.4%
Moderate or severe renal disease	3	4.3%
Psychiatric		
Depression	40	57.9%
Anxiety	41	59%
Bipolar disorder	29	42%
Schizophrenia	4	5.7%
ADHD	9	13%
Suicidal ideations	15	21.7%
Insomnia	20	29%
PTSD	18	26%
Borderline personality disorder	3	4.3%
Other	3	4.3%

*CVA*, cerebrovascular accident; *ADHD*, attention deficit hyperactivity disorder; *PTSD*, post-traumatic stress disorder.

**Table 3:** Predictors of treatment retention at one month and three months in a study of sublocade initiation in the emergency department.

Retention at 1 month		Chi-square	df	P		B	SE	P	Exp (B)	Exp(B) 95% CI for Exp (B)	

										Lower	Upper
Demographic variables	Housing Status	5.705	2	.06	Age	0.1	0.0	.10	1.1	1.0	1.1
					Housing status (stable/unstable)	0.8	0.7	.23	2.2	0.6	7.9
					Constant	−2.2	1.4	.13	0.1		
	Race	4.318	2	.12	Age	0.1	0.0	.05	1.1	1.0	1.1
					Race (White/non-White)	−0.2	0.6	.70	0.8	0.2	2.6
					Constant	−1.2	1.4	.38	0.3		
	Sex	7.516	2	.02	Age	0.1	0.0	.06	1.1	1.0	1.1
					Sex (M/F)	1.1	0.6	.07	2.9	0.9	9.3
					Constant	−3.2	1.7	.06	0.0		
Clinical variables		7.517	3	.06	Age	0.1	0.0	.03	1.1	1.0	1.2
					ED visits 6 months prior to index injection	−0.1	0.1	.13	0.9	0.8	1.0
					How many sublocade injections has the patient received before the index case? (not including the index injection)	0.2	0.2	.23	1.3	0.9	1.8
					Constant	−1.8	1.4	.19	0.2		
Comorbid diagnoses	Medical	4.217	2	0.12	Age	0.1	0.0	.06	1.1	1.0	1.1
					Medical comorbidity (y/n)	−0.1	0.6	.83	0.9	0.3	2.7
					Constant	−1.4	1.3	.28	0.2		
	Psychiatric	7.12	2	0.03	Age	0.1	0.0	.04	1.1	1.0	1.1
					Psychiatric comorbidity (y/n)	1.3	0.7	.08	3.6	0.8	15.0
					Constant	−2.7	1.6	.08	0.1		
	SUD				Age	0.1	0.0	.07	1.1	1.0	1.1
					SUD comorbidity (y/n)	2.0	0.7	< .001	7.5	1.8	31.8
					Constant	−3.1	1.6	.05	0.0		
Retention at 3 months											
Demographic variables	Housing Status	1.1	2	.59	Age	0.0	0.0	.35	1.0	1.0	1.1
					Housing status (stable vs unstable)	−0.3	0.5	.57	0.7	0.3	2.1
					Constant	0.1	1.2	.93	1.1	1.0	1.1
	Race	0.8	2	.66	Age	0.0	0.0	.37	1.0	0.3	2.5
					Race (White/non-White)	−0.2	0.6	.76	0.8		
					Constant	0.0	1.2	.98	1.0		
	Sex	1.0	2	.61	Age	0.0	0.0	.41	1.0	1.0	1.1
					Sex (M vs F)	0.3	0.5	.62	1.3	0.4	3.9
					Constant	−0.6	1.4	.67	0.5		
Clinical variables		6.4	3	.09	Age	0.0	0.0	.19	1.0	1.0	1.1
					ED visits 6 months prior to index injection	−0.1	0.1	.12	0.9	0.8	1.0
					How many sublocade injections has the patient received before the index case? (not including the index injection)	0.4	0.2	.10	1.5	0.9	2.3
					Constant	−0.8	1.3	.51	0.4		
Comorbid diagnoses	Medical	2.5	2	.29	Age	0.0	0.0	.35	1.0	1.0	1.1
					Medical comorbidity (y/n)	−0.7	0.5	.19	0.5	0.2	1.4
					Constant		0.0	1.0	1.0	1.0	
	Psychiatric	1.9	2	.38	Age	0.0	0.0	.36	1.0	1.0	1.1
					Psychiatric comorbidity (y/n)	0.8	0.7	.27	2.1	0.6	8.0
					Constant	−0.9	1.3	.50	0.4		
	SUD	6.1	2	.05	Age	0.0	0.0	.50	1.0	1.0	1.1
					SUD comorbidity (y/n)	1.6	0.7	.02	4.9	1.2	19.1
					Constant	−1.3	1.3	.30	0.3		

*CI*, confidence interval; df, degree of freedom ; *ED*, emergency department; *SE*, standard error; *SUD*, substance use disorder comorbidity.
